# Historic Redlining and Contemporary Behavioral Health Workforce Disparities

**DOI:** 10.1001/jamanetworkopen.2022.9494

**Published:** 2022-04-28

**Authors:** Clese E. Erikson, Randl B. Dent, Yoon Hong Park, Qian Luo

**Affiliations:** 1 Fitzhugh Mullan Institute for Health Workforce Equity, Department of Health Policy and Management, Milken Institute School of Public Health, The George Washington University, Washington, District of Columbia

## Abstract

This cross-sectional study investigates the association between redlining and behavioral health specialist supply in 2 mental health professional shortage areas.

## Introduction

As the nation continues to confront the lasting legacy of Jim Crow–era structural racism, attention is increasingly turning to the association between historical redlining policies and contemporary racial disparities in access to health care, including behavioral health.^1,2,3^ In the 1930s, the federally sponsored Home Owners’ Loan Corporation (HOLC) rated wealthier neighborhoods with predominantly White populations as low risk (designated as green or blue on maps) and neighborhoods with lower-income Black and immigrant populations as high risk (yellow or red), with red areas designated as a hazardous financial risk.^1^ While other forms of structural racism have undoubtedly contributed to ongoing disparities over the intervening decades, research now finds that redlining is associated with present-day delays in diagnosis and treatment of health conditions^2^ and poor mental health.^3^ However, no studies, to our knowledge, have examined variation in the supply of physicians or other behavioral health clinicians across HOLC-redlined neighborhoods.

This exploratory cross-sectional study used 2 counties designated as mental health professional shortage areas to investigate whether behavioral health specialists are equitably allocated across redlined neighborhoods in Richmond City County, Virginia (Richmond), and Guilford County, North Carolina (Greensboro). We compare clinician-to-population ratios and other neighborhood characteristics by HOLC grade to assess the association between redlining and current clinician supply, racial and ethnic demographics, and the area deprivation index measuring income, education, employment, and housing quality.^4^

## Methods

This cross-sectional study was approved by George Washington University's Institutional Review Board (IRB). Informed consent was waived by the IRB under 45 CFR §46.116 (d). Clinician address was obtained from 2019 IQVIA Xponent prescription data for *psychiatric specialists* (psychiatrists, child and adolescent psychiatrists, addiction psychiatrists, and addiction medicine specialists) and 2020 state licensure files on psychologists, licensed professional counselors, licensed clinical social workers, and licensed marriage and family therapists. Using the Google Maps Geocoding Application Programming Interface, a total of 765 clinician addresses were geocoded to 113 census block group areas in Richmond and 1178 clinicians to 189 census block groups in Greensboro; 141 out of 2734 records (5.16%) could not be geocoded. Using the Mapping Inequality shape files,^1^ a census block group was coded as green, blue, yellow, or red if its centroid was located in a corresponding HOLC-associated area. The 5-Year American Community Survey (2015-2019) provided census block–level population estimates for clinician to population calculations and percent self-reporting as American Indian or Alaska Native, Asian, Black, Native Hawaiian or other Pacific Islander, White, some other race, or 2 or more races and percent self-reporting as Hispanic or non-Hispanic for area demographics. Social deprivation indices were taken from the 2019 area deprivation index.^4^ We conducted analysis of variance and post hoc pairwise *t* tests to investigate differences between HOLC areas, with significance set at *P* < .05.

## Results

In Richmond, 32.94% of clinicians were located in HOLC-rated areas, and 19.69% of clinicians in Greensboro were located in these areas. Redlined areas were associated with lower per 1000 population counts of psychologists, counselors, and therapists compared with green HOLC areas (Richmond: 1.26 vs 3.72; Greensboro: 0.58 vs 13.35), lower counts of psychiatrists per 1000 population members (Greensboro: 0 vs 5.09), an increased percentage of Black individuals (Richmond: 71.47% vs 11.41%; Greensboro: 89.96% vs 7.77%), and higher mean area deprivation scores (Richmond: 71.8 vs 15.1; Greensboro: 93.4 vs 35.0) (Table and Figure).

**Table.** Clinician Supply and Population Demographics by HOLC Grade

Variable	Outcome, mean (SD)^a^				ANOVA *P* value	Green vs red *P* value^b^
	Green HOLC grade	Blue HOLC grade	Yellow HOLC grade	Red HOLC grade		
**Richmond, Virginia**						
Psychologists, counselors and therapists, No./1000 population members	3.72 (3.27)	3.46 (2.32)	2.16 (2.33)	1.26 (1.78)	<.001	.03
Psychiatric specialists, No./1000 population members	0	0.05 (0.20)	0.10 (0.25)	0.12 (0.40)	.70	NA
Population^c^	1181 (390)	1141 (508)	1416 (650)	1358 (643)	.46	NA
Race, %^c^						
American Indian or Alaska Native	0	0	0.08 (0.22)	0.07 (0.20)	.44	NA
Asian	1.76 (1.83)	1.87 (1.88)	1.89 (3.31)	1.94 (3.21)	1.00	NA
Black	11.41 (19.47)	26.96 (27.28)	49.03 (33.09)	71.47 (24.63)	<.001	<.001
Native Hawaiian or other Pacific Islander	0	0	0.01 (0.03)	0	.60	NA
White	83.43 (20.93)	68.27 (27.59)	45.07 (32.25)	22.07 (21.48)	<.001	<.001
Some other race	0.58 (1.09)	0.90 (1.46)	0.85 (1.41)	0.75 (.97)	.92	NA
≥2 Races	2.82 (2.90)	2.00 (1.93)	3.09 (3.01)	3.70 (3.74)	.42	NA
Ethnicity, %^c^						
Hispanic	0.96 (1.32)	1.47 (1.64)	4.17 (6.67)	2.03 (2.31)	.09	NA
Non-Hispanic	99.04 (1.32)	98.53 (1.64)	95.83 (6.67)	97.97 (2.31)	.09	NA
National ADI rank^d^	15.1 (11.3)	33.8 (11.5)	53.7 (28.3)	71.8 (20.0)	<.001	<.001
State ADI rank^d^	2.5 (1.4)	4.8 (1.3)	6.8 (3.0)	8.5 (1.6)	<.001	<.001
Census block groups, No.	10	14	27	28	NA	NA
**Greensboro, North Carolina**						
Psychologists, counselors, and therapists, No./1000 population members	13.35 (7.21)	12.36 (10.94)	3.14 (5.49)	0.58 (0.71)	<.001	.01
Psychiatric specialists, No./ per 1000 population	5.09 (9.56)	0.37 (0.90)	0.34 (1.25)	0	.04	.06
Population, No.^c^	897 (177)	1078 (268)	1361 (803)	1240 (380)	.46	NA
Race, %^c^						
American Indian or Alaska Native	0	0.69 (1.69)	0.32 (0.96)	0.47 (0.94)	.38	NA
Asian	0.55 (1.10)	3.99 (4.78)	4.44 (5.76)	0.09 (0.27)	.12	NA
Black	7.77 (8.23)	12.05 (8.55)	52.86 (21.73)	89.96 (7.45)	<.001	<.001
White	90.64 (8.33)	80.15 (14.38)	36.93 (19.88)	5.60 (4.67)	<.001	<.001
Some other race	0.46 (0.53)	0.80 (1.46)	2.51 (3.66)	0.63 (1.32)	.27	NA
≥2 Races	0.35 (0.71)	2.31 (2.40)	2.93 (2.85)	2.49 (2.50)	.36	NA
Ethnicity, %^c^						
Hispanic	2.92 (3.39)	2.44 (3.32)	7.58 (6.10)	5.86 (7.85)	.23	NA
Non-Hispanic	97.08 (3.39)	97.56 (3.32)	92.42 (6.10)	94.14 (7.85)	.23	NA
National ADI rank^d^	35.0 (11.5)	43.8 (6.2)	81.6 (15.8)	93.4 (4.7)	<.001	<.001
State ADI rank^d^	2.0 (0.8)	3.0 (0.6)	8.1 (2.3)	9.6 (0.5)	<.001	<.001
Census block groups, No.	4	6	20	8	NA	NA

Abbreviations: ADI, area deprivation index; ANOVA, analysis of variance; HOLC, Home Owners’ Loan Corporation; NA, not applicable.

^a^ This is the census block group–level mean outcome within each grade.

^b^ When the ANOVA tests were not statistically significant, with *P* value ≥ .05, post-hoc pairwise *t* tests were not conducted and therefore *P* values were reported as NA.

^c^ American Community Survey (2015-2019) for population and demographics.

^d^ ADI is a measure of social deprivation providing rankings of neighborhoods by socioeconomic disadvantage in a region of interest. It includes factors for the theoretical domains of income, education, employment, and housing quality.

**Figure.**
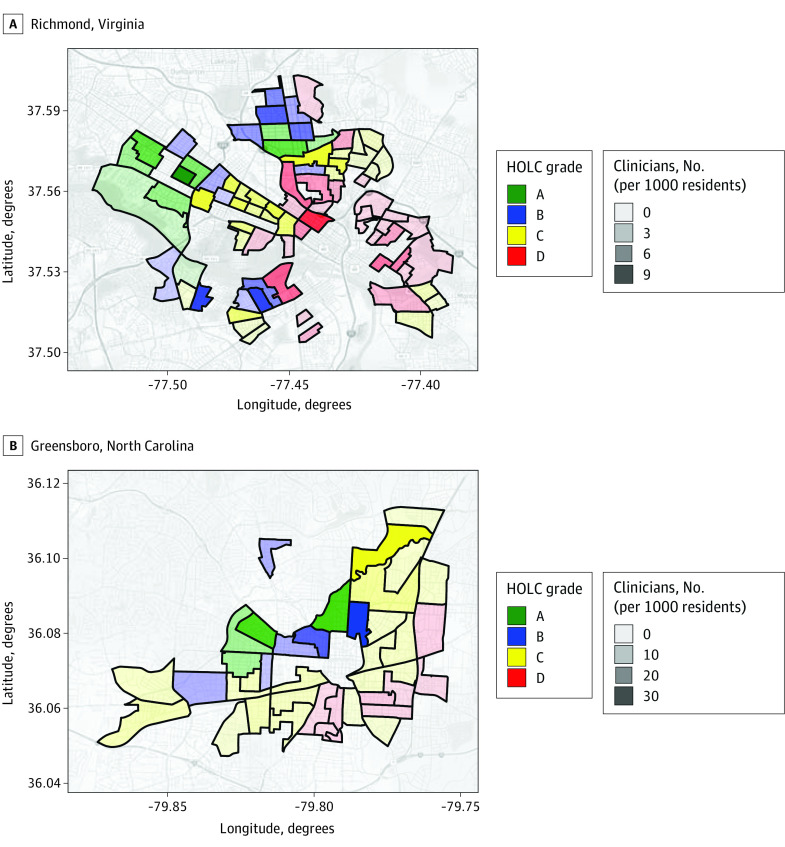
Clinicians per Capita by Home Owners’ Loan Corporation (HOLC) Grade Data sources were 2020 Virginia and North Carolina state licensure data on psychologists, licensed clinical social workers, licensed professional counselors, and licensed marriage and family therapists; Mapping Inequality shape files; and the American Community Survey (2015-2019). Darker shading indicates higher clinician counts per 1000 population members; green, highest-rated HOLC areas; red, lowest-rated HOLC areas.

## Discussion

This cross-sectional study’s preliminary findings suggest that structurally racist redlining policies from the 1930s were associated with decreased current behavioral health clinician availability in redlined communities. These results are consistent with recent research findings that redlining was associated with increased racial segregation and continued disinvestment^5^ and with health inequities.^2,3^ Transportation barriers compounded with greater behavioral health needs in redlined neighborhoods could make these workforce disparities even more stark than the data suggest.^3,6^ This study has several limitations, including that mailing address may not always reflect actual practice location, approximately 5% of records could not be geocoded, and results from 2 counties may not be generalizable to other redlined areas. These findings suggest that more research is needed on redlining and other historical and contemporary factors associated with racial and ethnic health inequities to inform federal policies, including health professional shortage area designations.
